# The genome sequence of the Wasp Spider, *Argiope bruennichi *(Scopoli, 1772)

**DOI:** 10.12688/wellcomeopenres.20339.1

**Published:** 2023-11-13

**Authors:** Liam M. Crowley, Finley Hutchinson, University of Oxford and Wytham Woods Genome Acquisition Lab, Darwin Tree of Life Barcoding collective, Wellcome Sanger Institute Tree of Life programme, Wellcome Sanger Institute Scientific Operations: DNA Pipelines collective, Tree of Life Core Informatics collective, Darwin Tree of Life Consortium

**Affiliations:** 1 University of Oxford, Oxford, England, UK; 2 University of Exeter, Penryn, England, UK

**Keywords:** Argiope bruennichi, Wasp Spider, genome sequence, chromosomal, Araneae

## Abstract

We present a genome assembly from an individual female *Argiope bruennichi* (the Wasp Spider; Arthropoda; Arachnida; Araneae; Araneidae). The genome sequence is 1,778.4 megabases in span. Most of the assembly is scaffolded into 13 chromosomal pseudomolecules, including the X _1_ and X _2_ sex chromosomes. The mitochondrial genome has also been assembled and is 14.06 kilobases in length.

## Species taxonomy

Eukaryota; Metazoa; Eumetazoa; Bilateria; Protostomia; Ecdysozoa; Panarthropoda; Arthropoda; Chelicerata; Arachnida; Araneae; Araneomorphae; Entelegynae; Orbiculariae; Araneoidea; Araneidae; *Argiope*; *Argiope bruennichi* (Scopoli, 1772) (NCBI:txid94029).

## Background

The Wasp Spider *Argiope bruennichi* (Scopoli, 1772) is a large and distinctive species of Araneid (orb-weaving) spider. The vernacular name refers to the black and yellow banding across the female’s abdomen which resembles the patterning of a social wasp. Several hypotheses have been suggested for the function of the female’s colouration, with Bush *et al.* (2008) concluding that it most likely evolved as a means of attracting insect prey to the spiders’ webs, as opposed to being a form of camouflage (by breaking up the spider’s outline). The same authors state that the possibility of aposematism playing a role in the evolution of the banding requires further study. Adult males are much smaller than adult females and lack the bold markings of the latter, instead having a pale brown abdomen with two vague, lighter longitudinal stripes.

Another intriguing characteristic of *A. bruennichi* (and other *Argiope* spp., which do not occur in the UK) is the zigzag stabilimentum, a pattern of thick silk which extends down from the centre of their webs. Like the spider’s pattern, this has led to many theories about its purpose. It was originally considered to have a stabilising function, although this view has been largely dismissed due to the loose attachment to the web ( Starks, 2002). The stabilimentum is formed from the same type of silk as the spider uses for wrapping up prey, and Walter *et al*. (2008) suggest it functions as a mechanism to maintain high silk gland activity, allowing more efficient ‘wrap attack’.

*Argiope bruennichi* was first recorded in the UK at Rye Harbour in 1922 and until the 1970s was very localised along the south coast of England, but has spread north and west rapidly since then ( Bee *et al.,* 2020). While most British records are still confined to the south-east, there are many records from Cornwall and it has now been recorded as far north as East Yorkshire ( British Arachnological Society, 2023). The species is characteristic of unmanaged grassland, wasteland and verges, spinning large orb webs in low vegetation. Orthoptera are the main prey, although a range of insects are taken. Adults peak in August and large, distinctive, urn-shaped egg-sacs are positioned higher in the vegetation with eggs overwintering ( Bee *et al.*, 2020).

The species has been extensively studied, with many papers published on various aspects of its biology and ecology. This is not the first publication dealing with its genomics: for example, Sheffer *et al*. (2021) published a chromosome-level reference genome for the species, and its complete mitochondrial genome ( Zhang *et al.*, 2016) and sex chromosomes ( Sheffer *et al.*, 2022) have also been sequenced.

We present a chromosomally complete genome sequence for *Argiope bruennichi*, using the standardised procedures set by the Darwin Tree of Life programme, ensuring consistency and comparability with other genomes sequenced within this project.

## Genome sequence report

**Figure 1.**
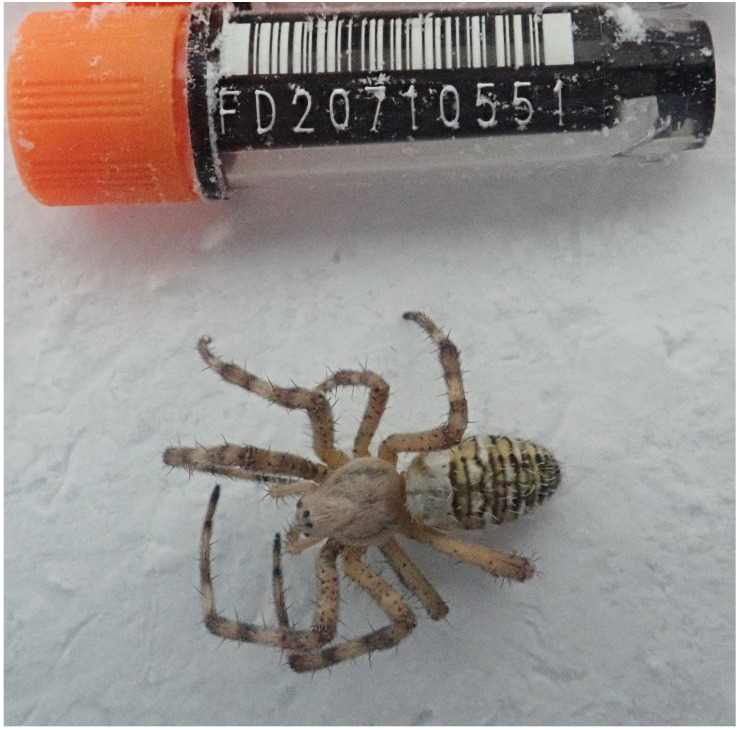
Photograph of the *Argiope bruennichi* (qqArgBrue1) specimen used for genome sequencing.

The genome was sequenced from one female *Argiope bruennichi* ( Figure 1) collected from Wytham Woods, Oxfordshire (biological vice-county Berkshire), UK (51.77, –1.34). A total of 28-fold coverage in Pacific Biosciences single-molecule HiFi long reads was generated. Primary assembly contigs were scaffolded with chromosome conformation Hi-C data. Manual assembly curation corrected 10 missing joins or mis-joins and removed 5 haplotypic duplications, reducing the assembly length by 0.25% and the scaffold number by 21.05%, and increasing the scaffold N50 by 2.39%.

**Figure 2.**
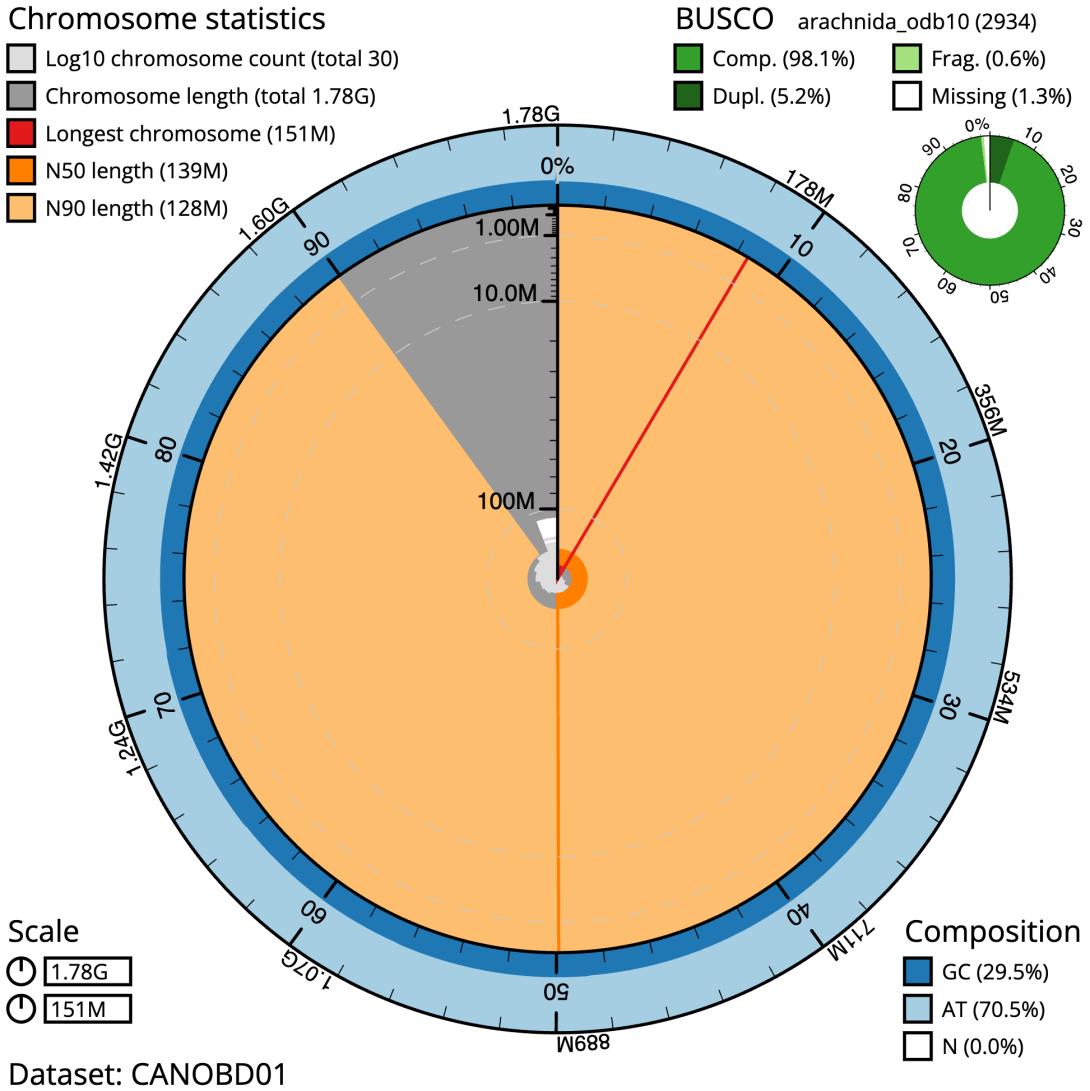
Genome assembly of *Argiope bruennichi*, qqArgBrue1.1: metrics. The BlobToolKit Snailplot shows N50 metrics and BUSCO gene completeness. The main plot is divided into 1,000 size-ordered bins around the circumference with each bin representing 0.1% of the 1,778,398,216 bp assembly. The distribution of scaffold lengths is shown in dark grey with the plot radius scaled to the longest scaffold present in the assembly (151,336,912 bp, shown in red). Orange and pale-orange arcs show the N50 and N90 scaffold lengths (139,055,214 and 127,865,338 bp), respectively. The pale grey spiral shows the cumulative scaffold count on a log scale with white scale lines showing successive orders of magnitude. The blue and pale-blue area around the outside of the plot shows the distribution of GC, AT and N percentages in the same bins as the inner plot. A summary of complete, fragmented, duplicated and missing BUSCO genes in the arachnida_odb10 set is shown in the top right. An interactive version of this figure is available at https://blobtoolkit.genomehubs.org/view/Argiope%20bruennichi/dataset/CANOBD01/snail.

**Figure 3.**
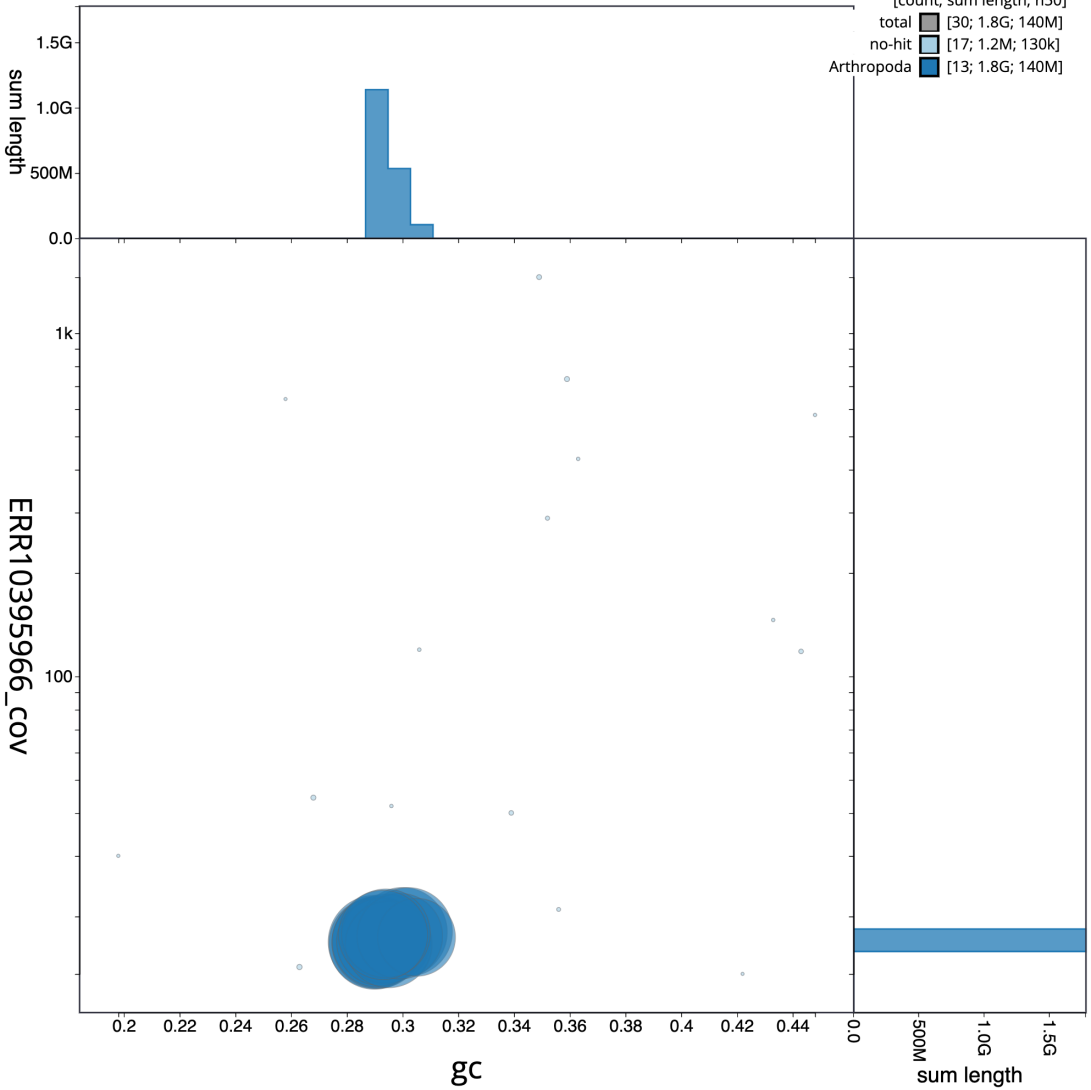
Genome assembly of *Argiope bruennichi*, qqArgBrue1.1: BlobToolKit GC-coverage plot. Scaffolds are coloured by phylum. Circles are sized in proportion to scaffold length. Histograms show the distribution of scaffold length sum along each axis. An interactive version of this figure is available at https://blobtoolkit.genomehubs.org/view/Argiope%20bruennichi/dataset/CANOBD01/blob.

**Figure 4.**
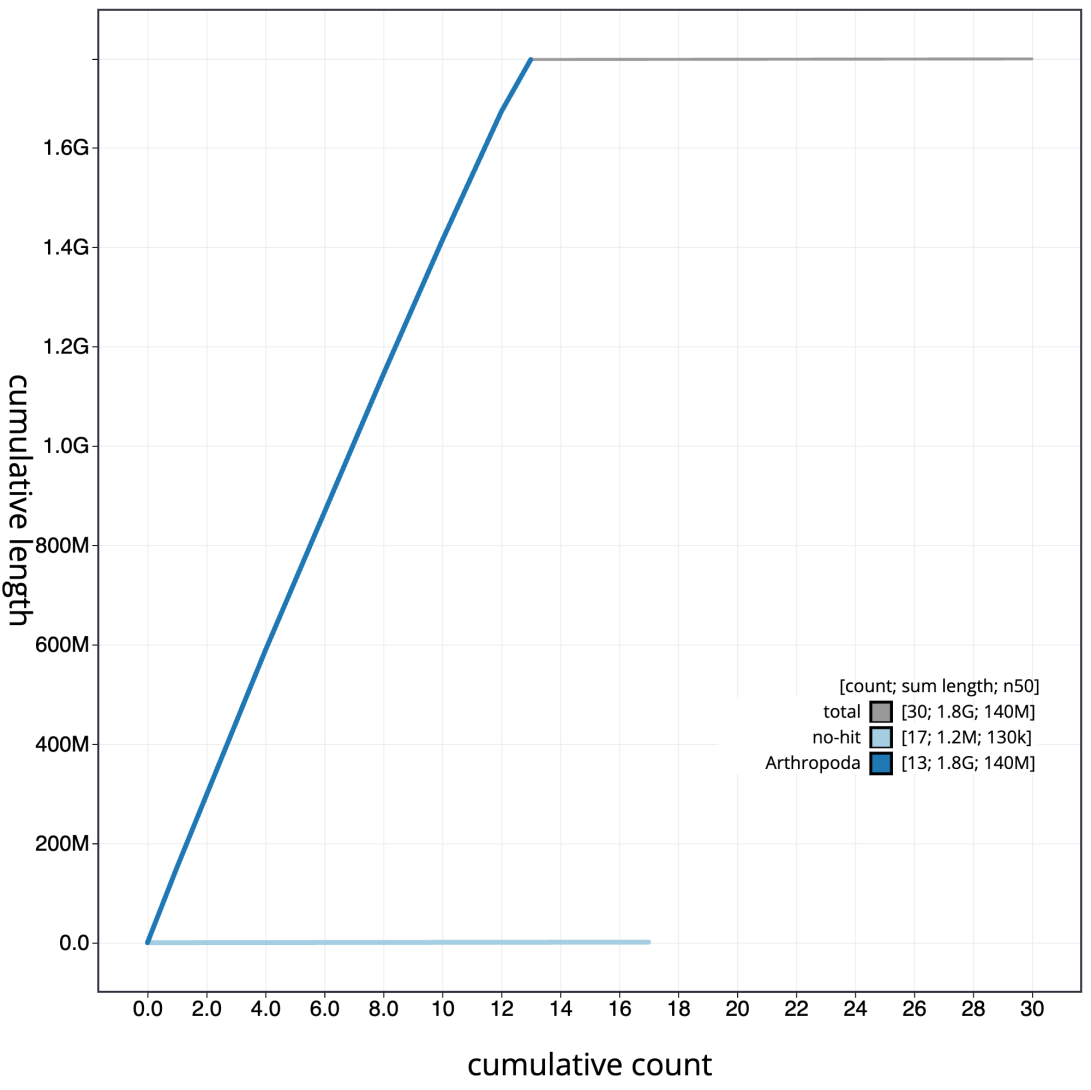
Genome assembly of *Argiope bruennichi*, qqArgBrue1.1: BlobToolKit cumulative sequence plot. The grey line shows cumulative length for all scaffolds. Coloured lines show cumulative lengths of scaffolds assigned to each phylum using the buscogenes taxrule. An interactive version of this figure is available at https://blobtoolkit.genomehubs.org/view/Argiope%20bruennichi/dataset/CANOBD01/cumulative.

**Figure 5.**
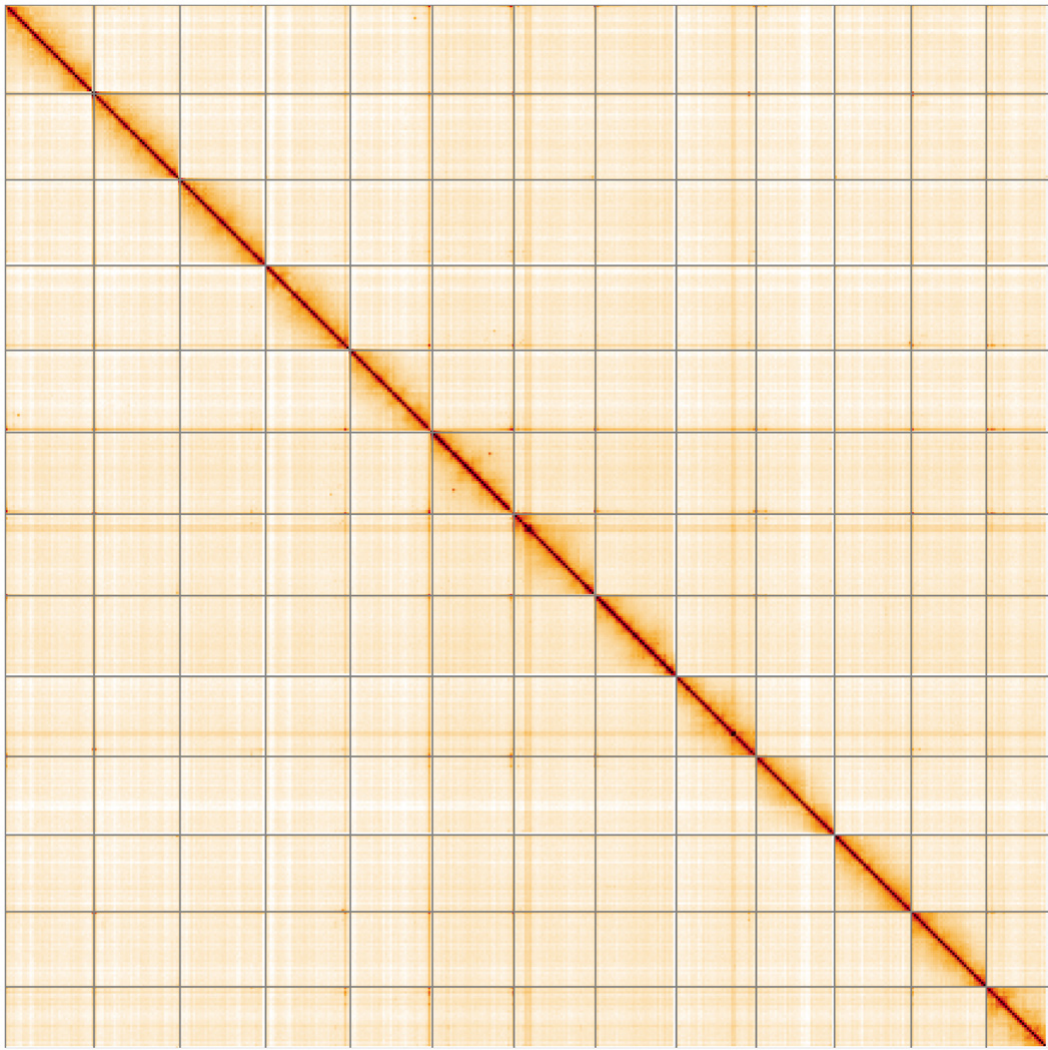
Genome assembly of *Argiope bruennichi*, qqArgBrue1.1: Hi-C contact map of the qqArgBrue1.1 assembly, visualised using HiGlass. Chromosomes are shown in order of size from left to right and top to bottom. An interactive version of this figure may be viewed at https://genome-note-higlass.tol.sanger.ac.uk/l/?d=e6_6dMh-TI-b_f7lmn0Zjg.

The final assembly has a total length of 1,778.4 Mb in 29 sequence scaffolds with a scaffold N50 of 139.1 Mb ( Table 1). A summary of the assembly statistics is shown in Figure 2, while the distribution of assembly scaffolds on GC proportion and coverage is shown in Figure 3. The cumulative assembly plot in Figure 4 shows curves for subsets of scaffolds assigned to different phyla. Most (99.93%) of the assembly sequence was assigned to 13 chromosomal-level scaffolds, representing 11 autosomes and the X _1_ and X _2_ sex chromosomes. The X _1_ and X _2_ chromosomes were determined based on alignment to a published assembly of *Argiope bruennichi* (GCA_015342795.1) ( Sheffer *et al.*, 2021). The sex chromosomes were identified in the published genome based on read mapping and cytology ( Sheffer *et al.*, 2022). Chromosome-scale scaffolds confirmed by the Hi-C data are named in order of size ( Figure 5; Table 2). While not fully phased, the assembly deposited is of one haplotype. Contigs corresponding to the second haplotype have also been deposited. The mitochondrial genome was also assembled and can be found as a contig within the multifasta file of the genome submission.

**Table 1.** Genome data for *Argiope bruennichi*, qqArgBrue1.1.

Project accession data		
Assembly identifier	qqArgBrue1.1	
Assembly release date	2022-12-13	
Species	*Argiope bruennichi*	
Specimen	qqArgBrue1	
NCBI taxonomy ID	94029	
BioProject	PRJEB56066	
BioSample ID	SAMEA10978988	
Isolate information	qqArgBrue1, female: cephalothorax (DNA sequencing and Hi-C data); abdomen (RNA sequencing)	
Assembly metrics *		*Benchmark*
Consensus quality (QV)	62.1	*≥ 50*
*k*-mer completeness	100%	*≥ 95%*
BUSCO **	C:98.1%[S:92.8%,D:5.2%], F:0.6%,M:1.3%,n:2934	*C ≥ 95%*
Percentage of assembly mapped to chromosomes	99.93%	*≥ 95%*
Sex chromosomes	X _1_, X _2_	*localised homologous pairs*
Organelles	Mitochondrial genome assembled	*complete single alleles*
Raw data accessions		
PacificBiosciences SEQUEL II	ERR10224932, ERR10395966	
Hi-C Illumina	ERR10297826	
PolyA RNA-Seq Illumina	ERR10908604	
Genome assembly		
Assembly accession	GCA_947563725.1	
*Accession of alternate haplotype*	GCA_947563775.1	
Span (Mb)	1,778.4	
Number of contigs	222	
Contig N50 length (Mb)	13.2	
Number of scaffolds	29	
Scaffold N50 length (Mb)	139.1	
Longest scaffold (Mb)	151.3	

* Assembly metric benchmarks are adapted from column VGP-2020 of “Table 1: Proposed standards and metrics for defining genome assembly quality” from ( Rhie *et al.*, 2021). ** BUSCO scores based on the $BUSCO_REF BUSCO set using v5.3.2. C = complete [S = single copy, D = duplicated], F = fragmented, M = missing, n = number of orthologues in comparison. A full set of BUSCO scores is available at https://blobtoolkit.genomehubs.org/view/Argiope%20bruennichi/dataset/CANOBD01/busco.

**Table 2.** Chromosomal pseudomolecules in the genome assembly of *Argiope bruennichi*, qqArgBrue1.

INSDC accession	Chromosome	Length (Mb)	GC%
OX387439.1	1	151.34	29.0
OX387440.1	2	146.54	29.0
OX387441.1	3	146.22	29.0
OX387442.1	4	144.11	29.0
OX387443.1	5	139.82	29.5
OX387445.1	6	139.06	30.0
OX387447.1	7	136.45	30.0
OX387448.1	8	133.23	29.0
OX387449.1	9	130.98	29.5
OX387450.1	10	127.87	30.0
OX387451.1	11	105.1	30.5
OX387446.1	X _1_	137.46	29.5
OX387444.1	X _2_	139.07	29.5
OX387452.1	MT	0.01	26.5

The estimated Quality Value (QV) of the final assembly is 62.1 with *k*-mer completeness of 100%, and the assembly has a BUSCO v5.3.2 completeness of 98.1% (single = 92.8%, duplicated = 5.2%), using the arachnida_odb10 reference set ( *n* = 2,934).

Metadata for specimens, barcode results, spectra estimates, sequencing runs, contaminants and pre-curation assembly statistics can be found at https://links.tol.sanger.ac.uk/species/94029.

## Methods

### Sample acquisition and nucleic acid extraction

A female *Argiope bruennichi* (specimen ID Ox001724, ToLID qqArgBrue1) was collected from Wytham Woods, Oxfordshire (biological vice-county Berkshire), UK (latitude 51.77, longitude –1.34) on 2021-07-28. The specimen was collected and identified by Liam Crowley (University of Oxford) and preserved on dry ice.

The workflow for high molecular weight (HMW) DNA extraction at the Wellcome Sanger Institute (WSI) includes a sequence of core procedures: sample preparation; sample homogenisation; DNA extraction; HMW DNA fragmentation; and fragmented DNA clean-up. The sample was prepared for DNA extraction at the WSI Tree of Life laboratory: the qqArgBrue1 sample was weighed and dissected on dry ice with tissue set aside for Hi-C sequencing ( https://dx.doi.org/10.17504/protocols.io.x54v9prmqg3e/v1). Tissue from the cephalothorax was disrupted using a Nippi Powermasher fitted with a BioMasher pestle ( https://dx.doi.org/10.17504/protocols.io.5qpvo3r19v4o/v1). DNA was extracted at the WSI Scientific Operations core using the Qiagen MagAttract HMW DNA kit, according to the manufacturer’s instructions.

RNA was extracted from abdomen tissue of qqArgBrue1 in the Tree of Life Laboratory at the WSI using the RNA Extraction: Automated MagMax™ *mir*Vana protocol ( https://dx.doi.org/10.17504/protocols.io.6qpvr36n3vmk/v1). The RNA concentration was assessed using a Nanodrop spectrophotometer and Qubit Fluorometer using the Qubit RNA Broad-Range (BR) Assay kit. Analysis of the integrity of the RNA was done using the Agilent RNA 6000 Pico Kit and Eukaryotic Total RNA assay.

All protocols developed by the Tree of Life laboratory are publicly available on protocols.io ( https://dx.doi.org/10.17504/protocols.io.8epv5xxy6g1b/v1).

### Sequencing

Pacific Biosciences HiFi circular consensus DNA sequencing libraries were constructed according to the manufacturers’ instructions. Poly(A) RNA-Seq libraries were constructed using the NEB Ultra II RNA Library Prep kit. DNA and RNA sequencing was performed by the Scientific Operations core at the WSI on Pacific Biosciences SEQUEL II (HiFi) and Illumina NovaSeq 6000 (RNA-Seq) instruments. Hi-C data were also generated from remaining cephalothorax tissue of qqArgBrue1 using the Arima2 kit and sequenced on the Illumina NovaSeq 6000 instrument.

### Genome assembly, curation and evaluation

Assembly was carried out with Hifiasm ( Cheng *et al.*, 2021) and haplotypic duplication was identified and removed with purge_dups ( Guan *et al.*, 2020). The assembly was then scaffolded with Hi-C data ( Rao *et al.*, 2014) using YaHS ( Zhou *et al.*, 2023). The assembly was checked for contamination and corrected as described previously ( Howe *et al.*, 2021). Manual curation was performed using gEVAL, HiGlass ( Kerpedjiev *et al.*, 2018) and Pretext ( Harry, 2022). The mitochondrial genome was assembled using MitoHiFi ( Uliano-Silva *et al.*, 2023), which runs MitoFinder ( Allio *et al.*, 2020) or MITOS ( Bernt *et al.*, 2013) and uses these annotations to select the final mitochondrial contig and to ensure the general quality of the sequence.

A Hi-C map for the final assembly was produced using bwa-mem2 ( Vasimuddin *et al.*, 2019) in the Cooler file format ( Abdennur & Mirny, 2020). To assess the assembly metrics, the *k*-mer completeness and QV consensus quality values were calculated in Merqury ( Rhie *et al.*, 2020). This work was done using Nextflow ( Di Tommaso *et al.*, 2017) DSL2 pipelines “sanger-tol/readmapping” ( Surana *et al.*, 2023a) and “sanger-tol/genomenote” ( Surana *et al.*, 2023b). The genome was analysed within the BlobToolKit environment ( Challis *et al.*, 2020) and BUSCO scores ( Manni *et al.*, 2021; Simão *et al.*, 2015) were calculated.

Table 3 contains a list of relevant software tool versions and sources.

**Table 3.** Software tools: versions and sources.

Software tool	Version	Source
BlobToolKit	4.1.7	https://github.com/blobtoolkit/blobtoolkit
BUSCO	5.3.2	https://gitlab.com/ezlab/busco
Hifiasm	0.16.1-r375	https://github.com/chhylp123/hifiasm
HiGlass	1.11.6	https://github.com/higlass/higlass
Merqury	MerquryFK	https://github.com/thegenemyers/MERQURY.FK
MitoHiFi	2	https://github.com/marcelauliano/MitoHiFi
PretextView	0.2	https://github.com/wtsi-hpag/PretextView
purge_dups	1.2.3	https://github.com/dfguan/purge_dups
sanger-tol/genomenote	v1.0	https://github.com/sanger-tol/genomenote
sanger-tol/readmapping	1.1.0	https://github.com/sanger-tol/readmapping/tree/1.1.0
YaHS	yahs-1.1.91eebc2	https://github.com/c-zhou/yahs

### Wellcome Sanger Institute – Legal and Governance

The materials that have contributed to this genome note have been supplied by a Darwin Tree of Life Partner. The submission of materials by a Darwin Tree of Life Partner is subject to the **‘Darwin Tree of Life Project Sampling Code of Practice’**, which can be found in full on the Darwin Tree of Life website here. By agreeing with and signing up to the Sampling Code of Practice, the Darwin Tree of Life Partner agrees they will meet the legal and ethical requirements and standards set out within this document in respect of all samples acquired for, and supplied to, the Darwin Tree of Life Project. 

Further, the Wellcome Sanger Institute employs a process whereby due diligence is carried out proportionate to the nature of the materials themselves, and the circumstances under which they have been/are to be collected and provided for use. The purpose of this is to address and mitigate any potential legal and/or ethical implications of receipt and use of the materials as part of the research project, and to ensure that in doing so we align with best practice wherever possible. The overarching areas of consideration are:

•   Ethical review of provenance and sourcing of the material

•   Legality of collection, transfer and use (national and international) 

Each transfer of samples is further undertaken according to a Research Collaboration Agreement or Material Transfer Agreement entered into by the Darwin Tree of Life Partner, Genome Research Limited (operating as the Wellcome Sanger Institute), and in some circumstances other Darwin Tree of Life collaborators.

## Data Availability

European Nucleotide Archive: *Argiope bruennichi* (wasp spider) Accession number PRJEB56066; https://identifiers.org/ena.embl/PRJEB56066 ( Wellcome Sanger Institute, 2022). The genome sequence is released openly for reuse. The *Argiope bruennichi* genome sequencing initiative is part of the Darwin Tree of Life (DToL) project. All raw sequence data and the assembly have been deposited in INSDC databases. The genome will be annotated using available RNA-Seq data and presented through the Ensembl pipeline at the European Bioinformatics Institute. Raw data and assembly accession identifiers are reported in Table 1.

## Author information

Members of the University of Oxford and Wytham Woods Genome Acquisition Lab are listed here: https://doi.org/10.5281/zenodo.7125292.

Members of the Darwin Tree of Life Barcoding collective are listed here: https://doi.org/10.5281/zenodo.4893703.

Members of the Wellcome Sanger Institute Tree of Life programme are listed here: https://doi.org/10.5281/zenodo.4783585.

Members of Wellcome Sanger Institute Scientific Operations: DNA Pipelines collective are listed here: https://doi.org/10.5281/zenodo.4790455.

Members of the Tree of Life Core Informatics collective are listed here: https://doi.org/10.5281/zenodo.5013541.

Members of the Darwin Tree of Life Consortium are listed here: https://doi.org/10.5281/zenodo.4783558.
