# Supplementary material for: The genome sequence of the Wasp Spider, Argiope bruennichi (Scopoli, 1772)
Source: Wellcome Open Res. 2023 Nov 13;8:522. [Version 1] doi: 10.12688/wellcomeopenres.20339.1 (PMC10904950; doi:10.12688/wellcomeopenres.20339.1)
Supplement: Peer review history

# Reviewer report: Reviewer response for version 1

**DOI:** 10.21956/wellcomeopenres.22522.r70616

**Published:** 2024-02-16

**Authors:** Jesper Bechsgaard

**Affiliations:** 1 Section for Genetic Ecology and Evolution, Department of Biology, Aarhus University, Aarhus, Denmark

The Data Note presents the genome sequencing of the spider species, *Argiope bruennichi*. Cutting edge technologies have been used to generate data and bioinformatic processing seem proper. RNA has been sequenced, but has not been used for the annotation of protein coding genes, which I find should have been done.

Are sufficient details of methods and materials provided to allow replication by others?

Partly

Is the rationale for creating the dataset(s) clearly described?

Partly

Are the datasets clearly presented in a useable and accessible format?

Yes

Are the protocols appropriate and is the work technically sound?

Yes

Reviewer Expertise:

population genetics and genomics

I confirm that I have read this submission and believe that I have an appropriate level of expertise to confirm that it is of an acceptable scientific standard.

# Reviewer report: Reviewer response for version 1

**DOI:** 10.21956/wellcomeopenres.22522.r70613

**Published:** 2023-12-04

**Authors:** Zhi-Sheng Zhang

**Affiliations:** 1 School of Life Sciences, Southwest University, Chongqing, Chongqing, China

This manuscript presents a genome assembly from an individual female *Argiope bruennichi* . The genome sequence is 1,778.4 megabases in span, and the assembly is scaffolded into 13 chromosomal pseudomolecules, including the X1 and X2 sex chromosomes. The mitochondrial genome has also been assembled. One more thing needs improvement: In Figure 5, the chromosome ID should be marked.

Are sufficient details of methods and materials provided to allow replication by others?

Yes

Is the rationale for creating the dataset(s) clearly described?

Yes

Are the datasets clearly presented in a useable and accessible format?

Yes

Are the protocols appropriate and is the work technically sound?

Yes

Reviewer Expertise:

Diversity and ecology of spiders from South China; Systematics, Phylogeny and biogeography of several spider groups, such as Lycosidae, Agelenidae, Dictynidae, Hahniidae, Amaurobiidae, Titanoecidae, etc.; Adapation of some spiders in different habitats; Spider silk.

I confirm that I have read this submission and believe that I have an appropriate level of expertise to confirm that it is of an acceptable scientific standard.

# Reviewer report: Reviewer response for version 1

**DOI:** 10.21956/wellcomeopenres.22522.r70614

**Published:** 2023-12-04

**Authors:** Sven Winter

**Affiliations:** 1 Research Institute of Wildlife Ecology, University of Veterinary Medicine Vienna, Vienna, Austria

The authors are presenting a high-quality reference genome for the wasp spider *Argiope bruennichi*, with most of the 1.7 Gb assembly length anchored into 13 chromosome-scale scaffolds, including the two sex chromosomes X1 and X2, and a BUSCO completeness of 98.1% complete BUSCOs. The assembly is an improvement over the previously published assembly (Sheffield *et al.*, 2021). 

 The rationale for generating this new assembly is not clearly stated. Yes, it is in improved assembly, but the report does not mention this. The only reason given is the consistency with the other Tree of Life genome assemblies. However, for me, this is not reason enough to generate a new assembly, as thousands of genomes of equally good quality are generated by other projects across the globe. I would have liked to see a comparison to the previously published assembly and a brief statement or discussion of what the new genome can provide to the scientific community that the other one did not already. 

 In addition, the methods are not sufficiently described for my liking. I am not a fan of publications that do not state the parameters used to generate the data but instead redirect the reader to a list of other publications or protocols to be reproducible. Especially as the bioinformatics protocols are somewhat hidden or unclear.

Are sufficient details of methods and materials provided to allow replication by others?

Partly

Is the rationale for creating the dataset(s) clearly described?

No

Are the datasets clearly presented in a useable and accessible format?

Yes

Are the protocols appropriate and is the work technically sound?

Yes

Reviewer Expertise:

genome assembly, sequencing,  population genomics

I confirm that I have read this submission and believe that I have an appropriate level of expertise to confirm that it is of an acceptable scientific standard, however I have significant reservations, as outlined above.
